# Flavonoids as Anxiolytics in Animal Tests: Systematic Review, Meta‐Analysis, and Bibliometrical Analysis

**DOI:** 10.1002/ptr.70060

**Published:** 2025-10-29

**Authors:** Jhennify Albuquerque Machado, Danilo Brandão Araújo, Monica Lima‐Maximino, Diógenes Henrique de Siqueira‐Silva, Bernardo Tomchinsky, Jonathan Cueto‐Escobedo, Juan Francisco Rodríguez‐Landa, Caio Maximino

**Affiliations:** ^1^ Laboratório de Neurociências e Comportamento “Frederico Guilherme Graeff” (LaNeC), Faculdade de Psicologia Universidade Federal do Sul e Sudeste do Pará (Unifesspa) Marabá Pará Brazil; ^2^ Laboratório de Neurofarmacologia e Biofísica (LaNeF) Universidade Do Estado Do Pará (UEPA) Marabá Pará Brazil; ^3^ Rede de Biodiversidade e Biotecnologia da Amazônia Legal (Rede Bionorte) Marabá Pará Brazil; ^4^ Grupo de Estudos da Reprodução de Peixes Amazônicos (GERPA), Faculdade de Biologia Universidade Federal do Sul e Sudeste do Pará (Unifesspa) Marabá Pará Brazil; ^5^ Laboratório de Botânica e Ecologia (Boteco), Faculdade de Biologia Universidade Federal do Sul e Sudeste do Pará (Unifesspa) Marabá Pará Brazil; ^6^ Instituto de Ciencias de la Salud, Universidad Veracruzana Xalapa Veracruz México; ^7^ Laboratorio de Neurofarmacología Instituto de Neuroetología, Universidad Veracruzana Xalapa Veracruz México; ^8^ Facultad de Química Farmacéutica Biológica Universidad Veracruzana Xalapa Veracruz México

**Keywords:** anxiolytics, ethnopharmacology, flavonoids, preclinical research, screening tests

## Abstract

Flavonoids are natural secondary metabolites of plants with a basic composition derived from polyphenols that can produce a plethora of different neurophysiological effects, some of which are relevant to anxiety disorders. As such, many flavonoids have been evaluated in behavioral screens in preclinical research on anxiolytics. We sought to map the potential of flavonoids as anxiolytics by bibliometric analysis and a systematic review and meta‐analysis of animal tests using these compounds. Bibliometric analysis suggests that the field is highly concentrated on a few research groups mostly located in the Global South, suggesting the need to improve international collaborations. The themes which emerged in the bibliometric analysis are driven by the exploratory steps of pharmacological research, including finding anxio‐selective effects and looking for dose–response patterns. This suggests that the field, as a whole, could benefit from more mechanistic and confirmatory research. The systematic review included 38 articles, with a total of *k* = 183 comparisons, comprising 43 different molecules. The meta‐analysis showed strong evidence for an anxiolytic‐like effect of flavonoids on animal tests, including assays made in rats, mice, and zebrafish (SMD = −1.457, 95% CI [−1.365 to −0.9264]). Subgroup analysis suggested that this effect is present in acute treatment (SMD = −1.0985 (95% CI: −1.31 to −0.88)), but not after chronic treatment (SMD = −1.96, 95% CI [−4.93; 1.01]). Study quality was overall moderate. We finish with a set of recommendations for preclinical research on the anxiolytic potential of flavonoids.

## 1 Introduction

Anxiety disorders such as generalized anxiety disorder, social anxiety disorder, and panic disorder are pathological states characterized by psychological and physical symptoms that severely deteriorate the quality of life of a great percentage of the population around the world (Yang et al. 2021). The first‐line treatments against anxiety include psychotherapy and pharmacotherapy based on drugs such as Selective Serotonin Reuptake Inhibitors (SSRIs) and Serotonin‐Norepinephrine Reuptake Inhibitors (SNRIs). However, benzodiazepines are still used in several countries despite their adverse effects such as pharmacological tolerance, withdrawal symptoms, dependence, sedation, and cognitive impairment (Cueto‐Escobedo et al. 2020; Gomez et al. 2018).

The need for new treatments without undesired side effects has led to the scientific research of secondary metabolites present in plants, which are first tested at the preclinical level using validated behavioral models of anxiety in animals, such as the Elevated Plus Maze (EPM), the Light–Dark Test (LDT), and the Open Field Test (OFT), among others used with laboratory rodents (Gencturk and Unal 2024), and the Novel Tank Test (NTT), the Light–Dark Test (LDT), and others used in the zebrafish (Gerlai 2023; Kysil et al. 2017). These tests show great utility as behavioral screens to explore the anxiolytic‐like effects of plant secondary metabolites, such as flavonoids (Cueto‐Escobedo et al. 2022; German‐Ponciano et al. 2018).

Flavonoids are natural secondary compounds of plants with a basic composition derived from polyphenols (hydroxyls attached to phenolic rings) with low molecular weight. Widely found in higher plants, there are around 6000 flavonoid molecules described in nature, which can be categorized into groups according to their chemical structures, size, and the degree of oxidation of the phenol, including chalcones, flavones, flavanones, flavonols, dihydroflavonols, isoflavones, anthocyanins, and aurones (Panche et al. 2016). In plants, they are synthesized through the shikimic acid or acetate (acetyl coenzyme A) metabolic pathways and the formation of a chalcone that is the precursor of all flavonoids. For plants, flavonoids have adaptive importance as antioxidants, in hormone production, communication, allelopathy, attracting pollinators and dispersers, protection against herbivory, antimicrobial activity, or protection against freezing (Samantha et al. 2011).

The presence of flavonoids in plants is related to genetic, environmental, and cultural (cultivation) factors, such as chemotypes, circadian cycles, phenology and maturation, part and structure of the plant, solar radiation, soil characteristics, water availability, altitude, temperature, among others. Another relevant factor in the production and availability of flavonoids, considering metabolic pathways, plant morphology and anatomy, environmental issues, and ecological functions, is the storage of these substances. They can be concentrated in fruits, flowers, barks, or in specialized structures such as trichomes, glands, and resin ducts (Samantha et al. 2011).

It is important to note that in each species, more than a dozen types of flavonoids are synthesized, usually with a predominance of a few that are used as molecular markers and are generally are those with the greatest pharmacological relevance. Considering the restricted occurrence of each group of secondary compounds in the plant kingdom, flavonoids are relevant molecules for studies in plant chemotaxonomy (Cronquist system) and pharmacology. Some groups of phenols are related to specific families or genera of plants (Alkin 2017).

Despite being found in low concentrations in plants, flavonoids have significant applications for the human population, such as dyes, food, nutraceuticals, cosmetics, flavorings, and medicines (Saini et al. 2024). They are the predominant component in many known medicinal plants (Alkin 2017). In pharmacological use, following the diversity of flavonoids, they are associated with a wide range of medical treatments as antioxidants, anti‐inflammatory, and anticancer drugs, also presenting effects on the central nervous system and in coronary diseases (Abou Baker 2022; Panche et al. 2016; Tungmunnithum et al. 2018), although their use as prescription drugs in Western conventional medicine is still lagging. Flavonoids are of special interest to psychopharmacology, given that they are found in medicinal plants with possible anxiolytic effects in different cultures, and some of these anxiolytic effects can be attributed to flavonoids (German‐Ponciano et al. 2018; Liu et al. 2021; López‐Rubalcava and Estrada‐Camarena 2016; Rodrigues et al. 2008). Experimental results show that flavonoids and their conjugate derivatives may cross the blood–brain barrier and exert pharmacological action on the central nervous system (Jäger and Saaby 2011; Youdim et al. 2003) by activating neuronal signaling pathways (Spencer 2007) and neurotransmitter systems (i.e., serotonergic, GABAergic, noradrenergic, and dopaminergic, among others) involved in neuropsychiatric disorders such as anxiety and depression (German‐Ponciano et al. 2018; Wang, Yang, et al. 2023; Wang, Zhao, et al. 2023).

There is considerable evidence that some naturally occurring flavonoids target the central benzodiazepine receptor (i.e., the benzodiazepine site at GABAA receptors) (Hanrahan et al. 2011; Marder et al. 2001). A pharmacophore model suggests that flavone derivatives bind the same site as benzodiazepines (BZD), due mainly to the negatively charged oxygen atom of the carbonyl group of the flavonoids and with the nature of the substituent in position 3′ (Marder et al. 2001). Kahnberg et al. (2002) produced a pharmacophore model with two additional regions of steric repulsive interactions between flavone ligands and the BZD site. Flavones, specifically, appear to create a range of biological activities at BZD sites, with some molecules acting as full positive allosteric modulators, others as partial positive allosteric modulators, and others as negative modulators (Hanrahan et al. 2011; Wasowski and Marder 2012). Interestingly, flavones such as apigenin and flavonone glycosides such as hesperidin target the GABAA/benzodiazepine receptor complex, producing anxiolytic‐like effects; however, depending on the doses, they may also produce sedative and sleep‐enhancing properties (Marder et al. 2003). Chrysin is another flavone that targets the GABAA/benzodiazepine receptor complex, producing anxiolytic‐like effects (Costa et al. 2025; Cueto‐Escobedo et al. 2020; Guillén‐Ruiz et al. 2024; Rodríguez‐Landa et al. 2019, 2021, 2022; Zanoli et al. 2000). However, differing from hesperidin and apigenin, this flavone is devoid of sedative and motor effects (Cueto‐Escobedo et al. 2020; Wolfman et al. 1994), an advantage compared with other flavonoids and benzodiazepines. This also highlights that flavonoids may differ in the neurochemical and neurotrophic pathways contributing to their anxiolytic‐like effects.

These effects of flavonoids suggest that this class of molecules can produce important effects on anxiety and defensive behavior. Indeed, a recent meta‐analysis of small clinical studies suggested that flavonoids produce significant anxiolytic effects (Jia et al. 2022). However, not only do flavonoids considerably differ in terms of their actions on the central BZD site (Hanrahan et al. 2011; Wasowski and Marder 2012), but these molecules also target different receptors and present receptor‐independent mechanisms such as reactive oxygen species (ROS) sequestration. Thus, the field of preclinical research on flavonoids as anxiolytic agents is wide, not only due to the diverse different possible mechanisms of action of specific molecule subclasses but also due to different research priorities within the field. The present work attempted to map this variation through both a bibliometric analysis of the field, trying to discern the conceptual and social structure of the field of preclinical research on flavonoids as anxiolytics, and a meta‐analysis of animal experiments on anxiolytic‐like effects of these molecules.

## 2 Methods

### 2.1 Bibliometric Analysis

#### 2.1.1 Data Collection and Screening

Data retrieval for bibliometric analysis was conducted on 18th May 2024, using PubMed (https://www.ncbi.nlm.nih.gov/pubmed) to search for articles describing the effects of flavonoids on anxiety‐like behavior in animal tests. The following string was used: ((((((((flavonoid[Title/Abstract]) AND (elevated plus‐maze[Title/Abstract])) OR ((flavonoid[Title/Abstract]) AND (light–dark test[Title/Abstract]))) OR ((flavonoid[Title/Abstract]) AND (open‐field test[Title/Abstract]))) OR ((flavonoid[Title/Abstract]) AND (holeboard[Title/Abstract]))) OR ((flavonoid[Title/Abstract]) AND (zebrafish[Title/Abstract]) AND (anxiety[Title/Abstract]))) OR (((flavonoid[Title/Abstract]) AND (rat[Title/Abstract]) AND (anxiety[Title/Abstract])))) OR (((flavonoid[Title/Abstract]) AND (mouse[Title/Abstract]) AND (anxiety[Title/Abstract])))) OR (((flavonoid[Title/Abstract]) AND (mice[Title/Abstract]) AND (anxiety[Title/Abstract]))). A search filter optimized for finding studies on animal experimentation on PubMed was used (Hooijmans et al. 2014). Only documents written in English were retained. A total of 197 documents were retained for bibliometric analysis.

#### 2.1.2 Bibliometric and Scientometric Analyses

Bibliometric analyses were made using the R package “bibliometrix” (Aria and Cuccurullo 2017). The package was used first to conduct a descriptive analysis, quantifying timespan, number of documents, annual scientific production, annual growth rate, document average age, average citations per document, description of most relevant sources and affiliations, and analyses of corresponding authors' countries. Influential sources were analyzed using Bradford's law (Bradford 1934/1985). The conceptual structure of the field was analyzed using co‐word analysis through keyword co‐occurrences; a network layout was generated using the Fruchterman–Reingold algorithm, plotting the main 50 cited references, with vertex similarities normalized using association strength. This network was described in terms of size, density, transitivity, degree of centralization, and average path length. A thematic map was drawn by crossing the density and centrality of keyword co‐occurrences, placing themes in four quadrants: (1) upper‐right quadrant: motor themes (themes that are both well‐developed and important for the structure of the specific research field); (2) lower‐right quadrant: basic themes (themes that are important for the research field but undeveloped); (3) lower‐left quadrant: emerging or disappearing themes (themes that are both weakly developed and with low external ties to other themes); (4) upper‐left quadrant: very specialized/niche themes (themes that have well‐developed internal ties but low external ties to other themes). Finally, the social structure of the field was analyzed using collaboration networks.

### 2.2 Meta‐Analysis

#### 2.2.1 Search Strategy

In order to find and filter data for the meta‐analysis, we searched PubMed (https://www.ncbi.nlm.nih.gov/pubmed) for articles describing the effects of flavonoids on anxiety‐like behavior in animal tests. The following string was used: ((((((((flavonoid[Title/Abstract]) AND (elevated plus‐maze[Title/Abstract])) OR ((flavonoid[Title/Abstract]) AND (light‐dark test[Title/Abstract]))) OR ((flavonoid[Title/Abstract]) AND (open‐field test[Title/Abstract]))) OR ((flavonoid[Title/Abstract]) AND (holeboard[Title/Abstract]))) OR ((flavonoid[Title/Abstract]) AND (zebrafish[Title/Abstract]) AND (anxiety[Title/Abstract]))) OR (((flavonoid[Title/Abstract]) AND (rat[Title/Abstract]) AND (anxiety[Title/Abstract])))) OR (((flavonoid[Title/Abstract]) AND (mouse[Title/Abstract]) AND (anxiety[Title/Abstract])))) OR (((flavonoid[Title/Abstract]) AND (mice[Title/Abstract]) AND (anxiety[Title/Abstract]))). A search filter optimized for finding studies on animal experimentation on PubMed was used (Hooijmans et al. 2014).

#### 2.2.2 Study Selection

The following inclusion criteria were used: studies that included primary behavioral data obtained from tests for anxiety‐like behavior in rats, mice, or zebrafish (light/dark test, novel tank test, elevated‐plus maze, open‐field test, or holeboard test); reporting of appropriate controls; reporting of at least sample sizes and summary statistics (mean and standard deviation or standard error of the mean), either in tables or graphs, for control and treated groups (da Silva Chaves et al. 2018). The exclusion criteria were: studies that evaluated the effects of non‐isolated flavonoids (e.g., mixtures of flavonoids or plant extracts), studies that only included abstracts, studies with incomplete text, studies published on non‐official websites, papers not in English, and duplicate publications. Whenever an experiment evaluated the effects of other drugs (e.g., GABAA receptor antagonists, to assess the role of these receptors in the effects of the flavonoid) or interventions (e.g., acute stress) on anxiety‐like behavior, only control and flavonoid groups were considered, and data under intervention effects were not analyzed. Possible confounds in relation to the role of development were reduced by excluding studies that were not performed on adult animals. Studies were screened by three reviewers from the author list, working independently based on the inclusion and exclusion criteria.

#### 2.2.3 Data Extraction

**FIGURE 1:**
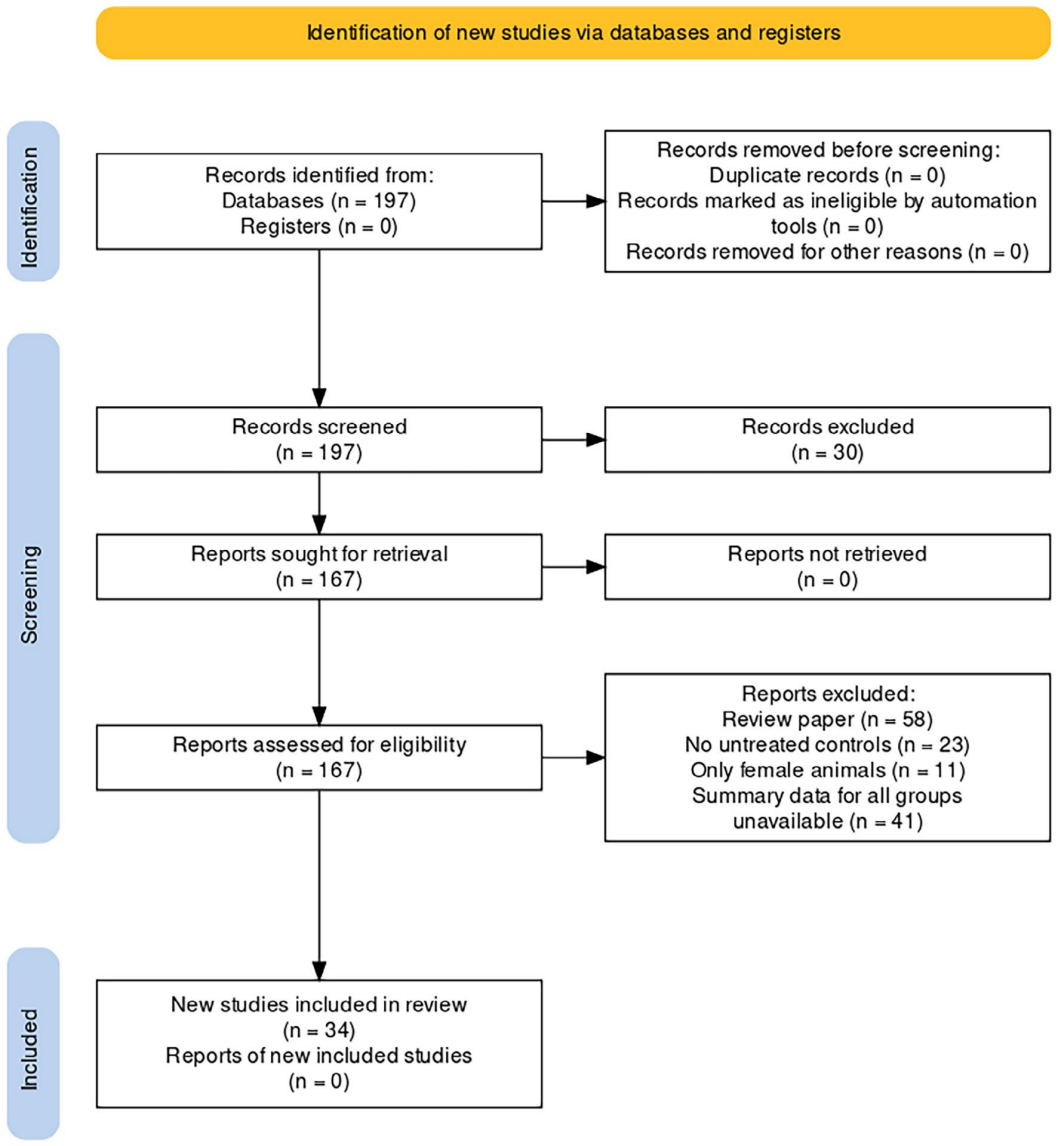
PRISMA flow diagram of the document screening process for the meta‐analysis.

The data retrieval and analysis flow can be found in Figure 1. The following data were extracted from each included study: identification (DOI, authors, publication year); species; strain/phenotype; behavioral test that was used; molecule; dose of the flavonoid; means and standard deviations for the primary outcome of each test (time on white/time on open arms/time on center or center:periphery ratio); and sample sizes (*N*) for each group. In studies in which data were graphically represented, means and standard deviations were extracted from figures using the PlotDigitizer online app (https://plotdigitizer.com/app). When multiple dependent variables were reported, only the primary endpoint was used. Following Vesterinen et al. (2014), when studies contained multiple comparisons with the control group (e.g., studies with multiple doses of the flavonoid of interest), the sample size for the control group was corrected for multiple comparisons by dividing the number reported by the number of treatment groups.

#### 2.2.4 Methodological Quality and Assessment of Studies

To assess the quality of the studies, SYRCLE's Risk of Bias tool was used (Hooijmans et al. 2014); briefly, the tool uses signaling questions to guide bias assessment in 10 domains (sequence generation, baseline characteristics, allocation concealment, random housing, blinding, random outcome assessment, blinding, incomplete outcome data, selective outcome reporting, and other sources of bias) divided into six general types of bias (selection bias, performance bias, detection bias, attrition bias, reporting bias, and other biases). Studies were rated using these signaling questions by at least two authors; ratings were assessed as “high risk,” “some concern,” “low risk,” “unclear risk,” and “not applicable.” Moreover, given the importance of using standardized extracts for experiment reproducibility (Izzo et al. 2016), a domain regarding the origin of the molecule was added. If compounds/molecules were acquired from certified vendors, “low risk” was attributed; if the article reported the steps for synthesis or isolation and analytical steps to analyze the molecule, “some concern” was attributed; articles that reported the use of fractions without isolation and purification were labeled as “high risk.” These risk of bias (RoB) ratings were assigned numbers (3 for high risk, 2 for unclear risk, 1 for low risk), and a median of all ratings for a single study was calculated; the median was then back‐transformed to the original ratings to construct an overall risk of bias rating for each study. Figures for RoB ratings were made on robvis (McGuinness and Higgins 2020).

#### 2.2.5 Data Analysis

A general meta‐analysis was made on all data, and subset analyses were made on data from different molecule classes and treatment durations; in the subset analyses, the procedures were the same as those used for the general meta‐analysis. The analysis was carried out using the standardized mean difference (SMDs) as the outcome measure (*yi*). Qualitative interpretations of the magnitudes of SMDs followed Cohen's (1988) guidelines. A random‐effects model was fitted to the data, which was displayed as forest plots. We also searched PubMed for information on binding affinities of flavonoid molecules for the GABAA receptor and analyzed the correlation between *yi* and the affinity *Ki*. The amount of heterogeneity (i.e., tau^2^) was estimated using the restricted maximum‐likelihood estimator (Viechtbauer 2005). In addition to the estimate of tau^2^, the *Q*‐test for heterogeneity (Cochran 1954) and the *I* ^2^ statistic were reported. In case any amount of heterogeneity is detected (i.e., tau^2^ > 0, regardless of the results of the *Q*‐test), a prediction interval for the true outcomes was also provided. Studentized residuals and Cook's distances were used to examine whether studies may be outliers and/or influential in the model context. The sources of heterogeneity were assessed via subgroup analyses, using treatment duration (acute vs. chronic) or molecule class. Studies with a studentized residual larger than the 95th percentile of a standard normal distribution were considered potential outliers (i.e., using a Bonferroni correction with two‐sided alpha = 0.05 for *k* comparisons included in the meta‐analysis). Studies with a Cook's distance larger than the median plus six times the interquartile range of the Cook's distances were considered to be influential. To analyze publication bias, the rank correlation test (Begg and Mazumdar 1994) and the regression test (Egger et al. 1997), using the standard error of the observed outcomes as predictors, were used to check for funnel plot asymmetry. Meta‐analyses were made using R (version 4.1.2), with the package “metafor” (version 3.8‐1; Viechtbauer 2010).

### 2.3 Dose–Response Curves

Dose–response curves for each of the molecule classes were obtained by fitting generalized log‐logistic 5 parameter models (LL5), with dose as an independent variable and observed effect size *yi* as the dependent variable—except for data on chalcones and flavanols since visual inspection of plots suggested an inverted‐U shaped curve; in this case, a 5‐parameter Brain‐Cousens model was fitted (Brain and Cousens 1989). To compare the general potency of molecule classes, effective doses (ED50) were calculated for each curve. Curves were estimated using R (version 4.1.2), with the package “drc” (version 3.0‐1; Ritz et al. 2015).

## 3 Results

### 3.1 Bibliometric Analysis

A total of 197 documents were retrieved for the analysis. The documents spanned 30 years, from 1994 to 2024. The annual growth rate of the field was 8.32% (Figure S1A), and the average document age was 6.6 years. Scientific production showed two peaks: between 2008 and 2010, and again following 2018 and 2019. The average number of co‐authors per document was 6.75, with an average of 0.182 documents per author. International co‐authorships comprised 17.26% of the documents. Except for the United States, which was the 7th most productive country in the field, the 10 most productive countries in the field were from the Global South (Figure S1B). A network of keyword co‐occurrences (Figure S1C) produced 960 nodes, with a density of 0.023, transitivity of 0.259, a low degree of centralization (0.399), and long average path lengths (2.421). The network revealed two main clusters: the green cluster, representing basic methods and mechanisms (serotonin metabolism, hippocampus); and the purple cluster, describing behavioral pharmacological methods. Thematic mapping (Figure S1D) suggested basic themes that describe basic methods (e.g., “behavior, animal/drug effects” or “dose‐response relationship, drug”); motor themes include methods related to phytochemistry and pharmacognosy (e.g., “chromatography, high‐pressure liquid” and “plant extracts/chemistry/pharmacology”); niche themes include molecular docking, signal transduction mechanisms, and neuropathology; and emerging themes include catechins and inflammation. An analysis of author collaboration networks suggested little collaboration outside of a given research group: the network was sparse (density of 0.007) and non‐centralized (centralization of 0.031), with low transitivity (0.787).

### 3.2 Meta‐Analysis

The search parameters retrieved 206 articles in the PubMed database, of which 38 were retained for analysis after the application of inclusion criteria (Tables 1 and S1). A total of *k* = 183 comparisons were included in the analysis; this is because most articles included more than one dose and/or more than one flavonoid. The smallest number of comparisons in a single article was 1, and the largest was 16. A total of 43 different molecules were included in the analysis (Table 1). Results of the RoB assessment are presented in Figure S2. None of the studies were judged to be of critical RoB, and therefore all studies were included in posterior analyses. Nonetheless, most studies were assessed to have a RoB above the rating of low, and the vast majority had a rating of “unclear” in critical areas, such as randomization and blinding.

**TABLE 1:** Studies included in the meta‐analysis, including molecule class, specific molecules under the class, total number of comparisons included in the analysis (*k*), proportion of the total comparisons comprised by that molecule, and the reference.

Class	Molecule	Counts (*k*)	Total %	Studies	*K*_*i*_ for GABAA receptors, μM (ref.)
Flavones (10) 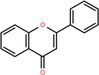	2′‐methoxy‐6‐methylflavone	8	5%	Karim et al. (2012)	NA
	3‐hydroxy‐2‐methoxy‐6‐methylflavone	8	5%	Karim et al. (2011)	NA
	5‐methoxyflavone	6	4%	Shanmugasundaram et al. (2020)	NA
	Apigenin	7	4%	Avallone et al. (2000); Viola et al. (1995); Zanoli et al. (2000)	4 (Wang et al. 2005)
	Chrysin	12	4%	Costa et al. (2025); German‐Ponciano et al. (2020); Wolfman et al. (1994)	3 (Wang et al. 2005)
	Flavone	1	1%	German‐Ponciano et al. (2020)	1 (Marder et al. 1996)
	Wogonin	4	2%	Hui et al. (2002)	0.92 (Hui et al. 2002)
	6‐methylapigenin	2	1%	Marder et al. (2003)	0.495 (Wasowski et al. 2002)
	Luteolin	4	2%	Coleta et al. (2008)	60.1 (Coleta et al. 2008)
	5,7,2′‐trihydroxy‐6,8‐dimethoxyflavone	1	1%	Huen et al. (2003)	0.0061 (Huen et al. 2003)
Chalcones (16) 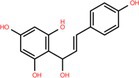	Butein	2	1%	Jamal et al. (2008)	NA
	Isoliquiritigenin	2	1%	Jamal et al. (2008)	0.45 (Cho et al. 2011)
	2′,2‐Dihydroxychalcone	2	1%	Jamal et al. (2008)	NA
	2′‐Hydroxy‐3,4‐Dimethoxychalcone	2	1%	Jamal et al. (2008)	NA
	4′,4‐Dichlorochalcone	2	1%	Jamal et al. (2008)	NA
	1,3‐bis (4‐chlorophenyl)‐3‐(carboxy‐methyl‐thio) prop‐an‐1‐one	2	1%	Jamal et al. (2008)	NA
	4′‐Chloro‐4‐methoxy‐chalcone	2	1%	Jamal et al. (2008)	NA
	1‐(4‐Chlorophenyl)‐3 (4‐methoxy‐phenyl)‐3‐(carboxymethylthio) prop‐an‐1‐one	2	1%	Jamal et al. (2008)	NA
	(E)‐3‐(2,4‐dichlorophenyl)‐1‐(2‐hydroxy‐3,4,6‐trimethoxyphenyl)prop‐2‐en‐1‐one	3	2%	Ferreira et al. (2021)	NA
	(E)‐3‐(4‐Chlorophenyl)‐1‐(2‐hydroxy‐3,4,6‐trimethoxyphenyl)prop‐2‐en‐1‐one	3	2%	Ferreira et al. (2021)	NA
	(E)‐3‐(2‐Fluorophenyl)‐1‐(2‐hydroxy‐3,4,6‐trimethoxyphenyl)prop‐2‐en‐1‐one	3	2%	Ferreira et al. (2021)	NA

	(E)‐3‐(4‐Fluorophenyl)‐1‐(2‐hydroxy‐3,4,6‐trimethoxyphenyl)prop‐2‐en‐1‐one	3	2%	Ferreira et al. (2021)	NA
	(2E, 4E)‐1‐(2‐hydroxy‐3,4,6‐trimethoxyphenyl) − 5‐phenylpenta‐2,4‐dien‐1‐one	3	2%	Xavier et al. (2020)	NA
	4′‐[(2E)‐3‐(3‐nitrophenyl)‐1‐(phenyl) prop‐2‐en‐1‐one] acetamide	3	2%	Ferreira et al. (2020)	NA
	(E)‐3‐(4‐(dimethylamino)phenyl)‐1‐(2‐hydroxyphenyl)prop‐2‐en‐1‐one	6	4%	Santos Oliveira et al. (2024)	NA
N‐{(4'‐[(E)‐3‐(4‐fluorophenyl)‐1‐(phenyl) prop‐2‐en‐1‐one]} acetamide	3	2%	Ferreira et al. (2019)	NA
Flavonols (4) 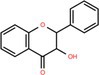	Kaempferol	12	7%	Grundmann et al. (2009); Viola et al. (1994); Vissiennon et al. (2012)	NA
	Quercetin	7	4%	Vissiennon et al. (2012)	NA
	Myricetin	4	2%	Vissiennon et al. (2012)	NA
	Fisetin	1		da Silva et al. (2024)	NA
Flavanones (3) 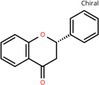	Naringenin	7	4%	Anderson et al. (2012); Nachammai et al. (2021)	2000 (Jäger et al. 2007)
	Naringin	9	5%	Ben‐Azu et al. (2018)	NA
	6‐methoxyflavanone	4	2%	Akbar et al. (2017)	NA
Flavanols (1) 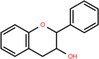	(−)‐epigallocatechin gallate	4	2%	Vignes et al. (2006)	62 (Hossain et al. 2002)
Isoflavones (1) 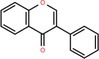	Puerarin	3	2%	Qiu et al. (2018)	18.46 (Wang et al. 2005)
Glycoside derivatives (4)	Quercitrin	6	4%	Li et al. (2016)	NA
	Rutin	7	4%	Anesti et al. (2020); Hernandez‐Leon et al. (2017)	NA
	Spinosin	6	4%	Liu et al. (2015)	NA
	Robinin	1		Lemos et al. (2025)	NA
Glucoside derivatives (3)	Apigenin 7‐glucoside	3	2%	Kumar and Bhat (2014)	NA
	Hesperidin	2	1%	Marder et al. (2003)	NA
	Tricin‐7‐O‐glucoside	1		Castolo‐Sanchez et al. (2024)	NA
Flavonolignans (1)	Silibinin	6	4%	Nachammai et al. (2021)	NA

*Note:* A single study can count a large number of comparisons due to the use of a larger number of doses.

For the overall meta‐analysis, the observed standardized mean differences ranged from −20.1471 to 12.9258, with the majority of estimates (87%) being negative in relation to control groups (i.e., suggesting decreased anxiety‐like behavior). The estimated average standardized mean difference based on the random‐effects model was SMD = −1.457 (95% CI: −1.365 to −0.9264) (Figure S3), interpreted as a large effect size. Therefore, the average outcome differed significantly from zero (*z* = −10.2393, *p* < 0.0001). According to the *Q*‐test, the true outcomes appear to be heterogeneous (*Q*[df = 182] = 611.3753, *p* < 0.0001, *τ* ^2^ = 1.3252, *I* ^2^ = 68.9%). A 95% prediction interval for the true outcomes is given by −4.7318 to 1.8521. Hence, although the average outcome is estimated to favor the flavonoid, in some studies the true outcome may favor the control. An examination of the studentized residuals revealed that several comparisons (the effect of the highest dose of hesperidin on the holeboard in mice in Marder et al. (2003); the effect of the highest dose of silibinin and naringenin on the novel tank test and of the lowest doses of both molecules on the light/dark test in zebrafish in Nachammai et al. (2021)) had values larger than ±3.6204 and may be potential outliers in the context of this model. According to the Cook's distances, several comparisons (the effects of all doses of silibinin on the novel tank test and of the highest dose of this molecule on the light/dark test in zebrafish in Nachammai et al. (2021)) could be overly influential. The rank correlation and the regression tests indicated potential funnel plot asymmetry (*p* < 0.001 for all tests; Figure S4), suggesting publication bias toward positive results. A small number of comparisons (*k* = 43) involved molecules with recorded *Ki* values; no significant correlation between the inverse of *Ki* for the GABAA receptor and log(*yi*) was found (Pearson's product–moment correlation = −0.079, *p* = 0.6121).

#### 3.2.1 Subgroup Analysis: Treatment Duration

Both acute (*k* = 161) and chronic (*k* = 22) treatment regimens were found in the literature search. Acute treatments (Figure S5) appeared to produce consistent anxiolytic‐like effects; the estimated average standardized mean difference based on the random‐effects model was SMD = −1.0985 (95% CI: −1.31 to −0.88), suggesting large effects. The average outcome significantly differed from zero (*z* = −10.09, *p* < 0.0001). According to the *Q*‐test, the true outcomes appear to be moderately heterogeneous (*Q*[df = 160] = 447.181, *p* < 0.0001, *τ* ^2^ = 1.031, *I* ^2^ = 63.51%).

Chronic treatment, on the other hand, did not produce either anxiolytic‐ or anxiogenic‐like effects (Figure S6). The estimated average standardized mean difference based on the random‐effects model was SMD = −1.96 (95% CI: −4.93 to 1.01). The average outcome did not significantly differ from zero (*z* = −1.29, *p* = 0.195). According to the Q‐test, the true outcomes appear to be highly heterogeneous (*Q*[df = 21] = 162.96, *p* < 0.0001, *τ* ^2^ = 46.21, *I* ^2^ = 98.55%).

#### 3.2.2 Subgroup Analyses: Molecule Class

**FIGURE 2:**
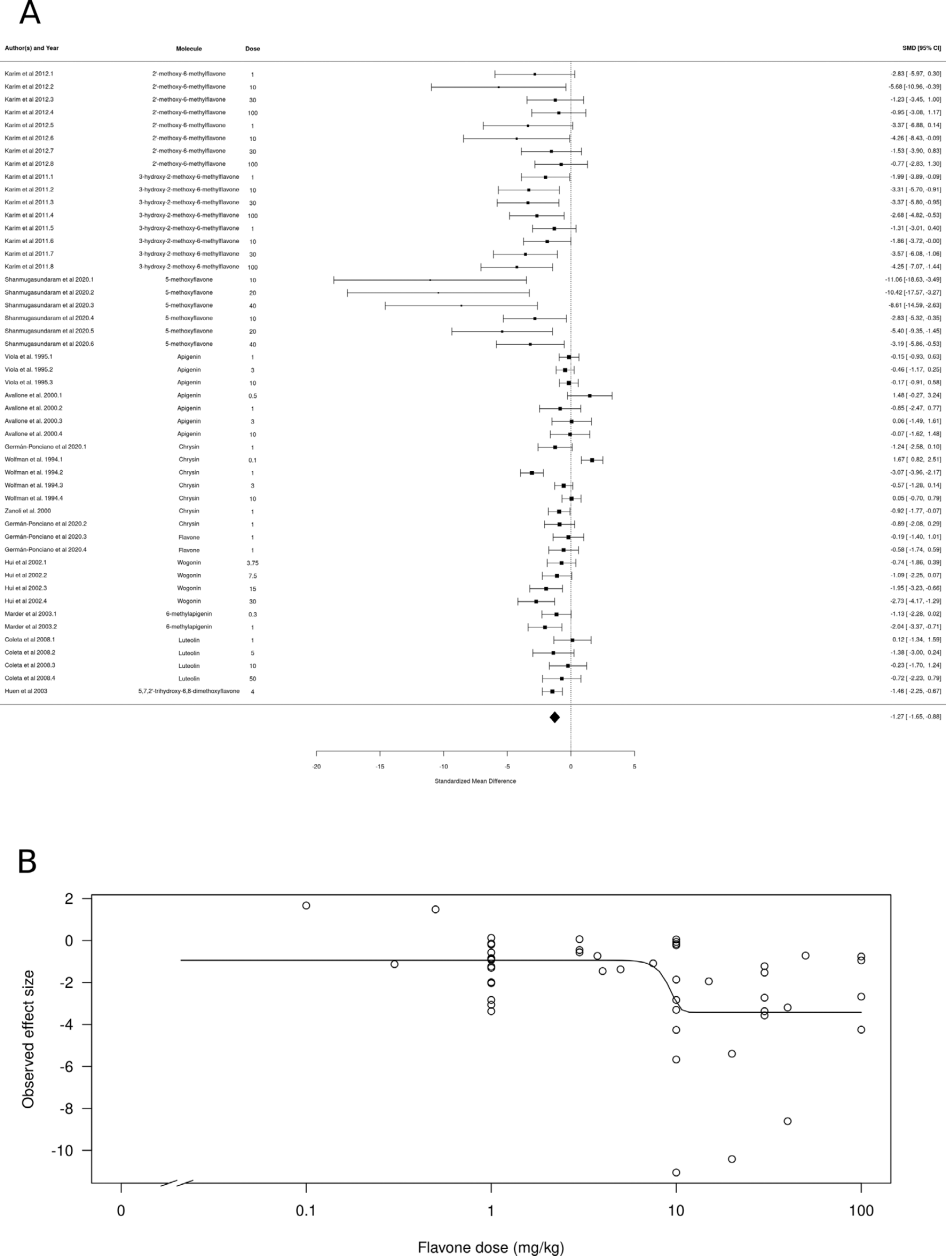
Subgroup analysis for flavones. (A) Forest plot showing the results of 54 comparisons examining the effect of a flavone on anxiety‐like behavior in animal tests. The figure shows the standardized mean difference (SMD) between control and flavone‐treated groups with corresponding 95% confidence intervals in the individual comparison, based on a random‐effects model. A negative standardized mean difference (SMD) corresponds to decreased anxiety‐like behavior, while a positive SMD corresponds to increased anxiety‐like behavior after flavone treatment. The overall effect size is denoted by the diamond symbol. (B) Estimated dose–response curve, based on the observed effect size (SMD) with increasing flavone dose. Each point corresponds to a single comparison from the forest plot. The curve was obtained by fitting generalized log‐logistic 5‐parameter models, with dose as an independent variable and observed effect size SMD as a dependent variable.

For flavones (*k* = 54), the estimated average standardized mean difference based on the random‐effects model was SMD = −1.16 (95% CI: −1.54 to −0.77) (Figure 2A), interpreted as a large effect size. Therefore, the average outcome differed significantly from zero (*z* = −5.864, *p* < 0.0001), suggesting an overall anxiolytic‐like effect. According to the *Q*‐test, the true outcomes appear to be heterogeneous (*Q*[df = 53] = 200.08, *p* < 0.0001, *τ* ^2^ = 1.29, *I* ^2^ = 73.61%). There is some evidence for a dose‐dependent effect, with higher doses producing larger effects (Figure 2B), with a calculated ED50 of 0.55 ± 0.25 mg/kg (mean ± SEM).

**FIGURE 3:**
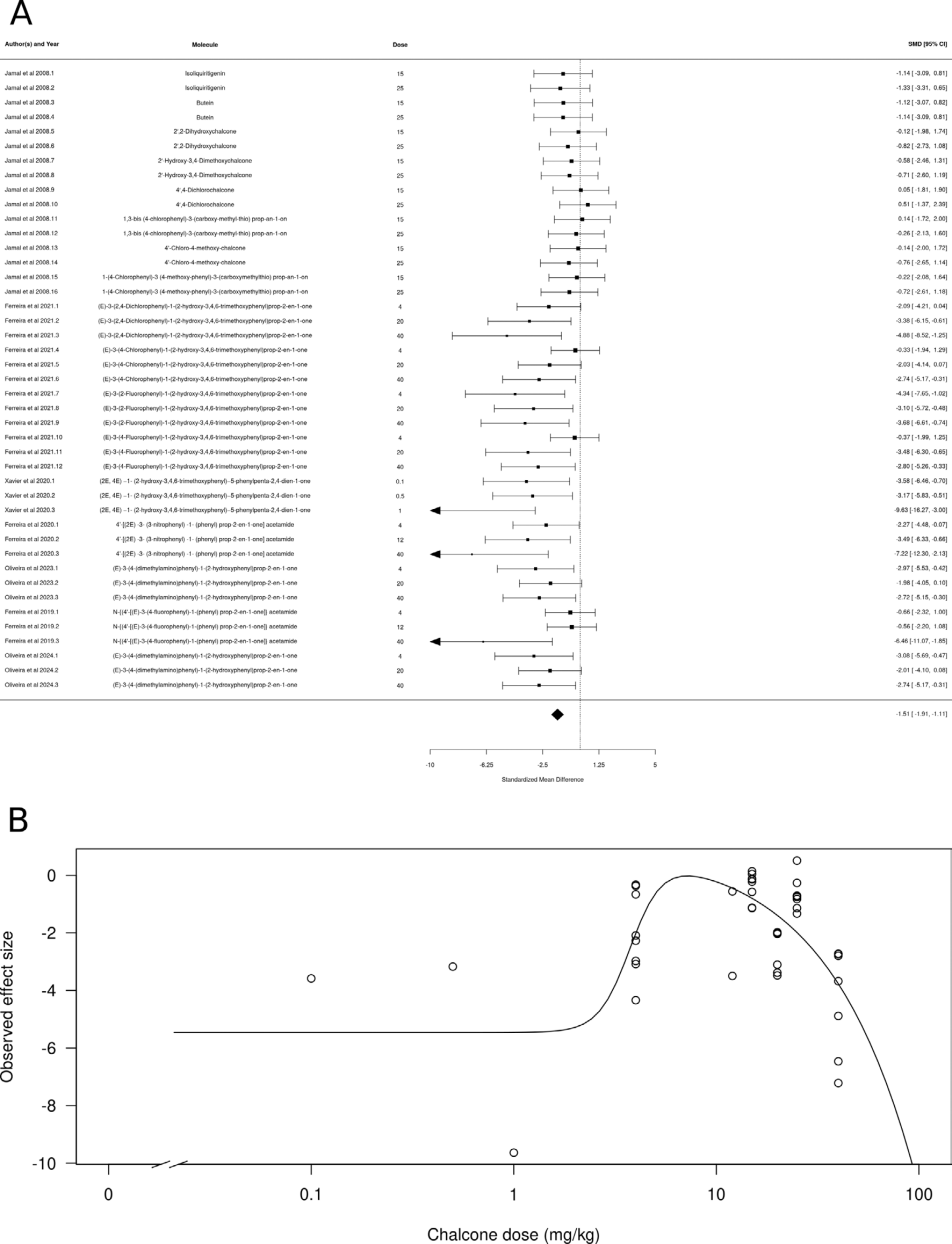
Subgroup analysis for chalcone. (A) Forest plot showing the results of 43 comparisons examining the effect of a chalcone on anxiety‐like behavior in animal tests. The figure shows the standardized mean difference (SMD) between control and chalcone‐treated groups with corresponding 95% confidence intervals in the individual comparison, based on a random‐effects model. A negative standardized mean difference (SMD) corresponds to decreased anxiety‐like behavior, while a positive SMD corresponds to increased anxiety‐like behavior after chalcone treatment. The overall effect size is denoted by the diamond symbol. (B) Estimated dose–response curve, based on the observed effect size (SMD) with increasing chalcone dose. Each point corresponds to a single comparison from the forest plot. The curve was obtained by fitting a 5‐parameter Brain–Cousens model, with dose as an independent variable and observed effect size SMD as a dependent variable.

Similar results were found for chalcones (*k* = 43), in which the estimated average standardized mean difference based on the random‐effects model was SMD = −1.51 (95% CI: −1.91 to −1.11) (Figure 3A), interpreted as a large effect size. Therefore, the average outcome differed significantly from zero (*z* = −7.4591, *p* < 0.0001), suggesting an overall anxiolytic‐like effect. According to the *Q*‐test, the true outcomes appear to be heterogeneous (Q[df = 42] = 69.1009, *p* = 0.005, *τ* ^2^ = 0.478, *I* ^2^ = 28.15%). There is evidence for a dose‐dependent effect, with smaller and larger doses producing larger effects (Figure 3B), with a calculated ED50 of 27.127 ± 5.74 mg/kg (mean ± SEM).

**FIGURE 4:**
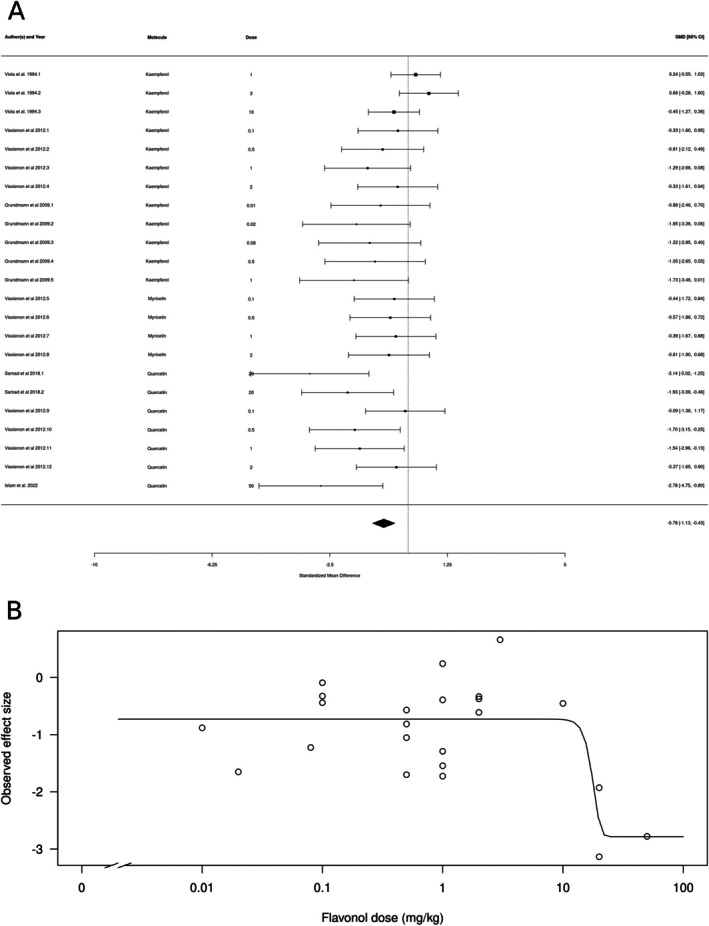
Subgroup analysis for flavonols. (A) Forest plot showing the results of 23 comparisons examining the effect of a flavonol on anxiety‐like behavior in animal tests. The figure shows the standardized mean difference (SMD) between control and flavonol‐treated groups with corresponding 95% confidence intervals in the individual comparison, based on a random‐effects model. A negative standardized mean difference (SMD) corresponds to decreased anxiety‐like behavior, while a positive SMD corresponds to increased anxiety‐like behavior after flavonol treatment. The overall effect size is denoted by the diamond symbol. (B) Estimated dose–response curve, based on the observed effect size (SMD) with increasing flavonol dose. Each point corresponds to a single comparison from the forest plot. The curve was obtained by fitting generalized log‐logistic 5‐parameter models, with dose as an independent variable and observed effect size SMD as a dependent variable.

Flavonols (*k* = 23) also appeared to produce an anxiolytic‐like effect (Figure 4A). The estimated average standardized mean difference based on the random‐effects model was SMD = −1.39 (95% CI: −1.13 to −0.42), interpreted as a medium‐to‐large effect size. Therefore, the average outcome differed significantly from zero (*z* = −4.3281, *p* < 0.0001), suggesting an overall anxiolytic‐like effect. According to the *Q*‐test, the true outcomes appear to be heterogeneous (*Q*[df = 22] = 36.3998, *p* = 0.0275, *τ* ^2^ = 0.2743, *I* ^2^ = 38.9%). There is some evidence for a dose‐dependent effect, with higher doses producing larger effects (Figure 4B), and a calculated ED50 of 17.563 ± 7.08 mg/kg (mean ± SEM).

**FIGURE 5:**
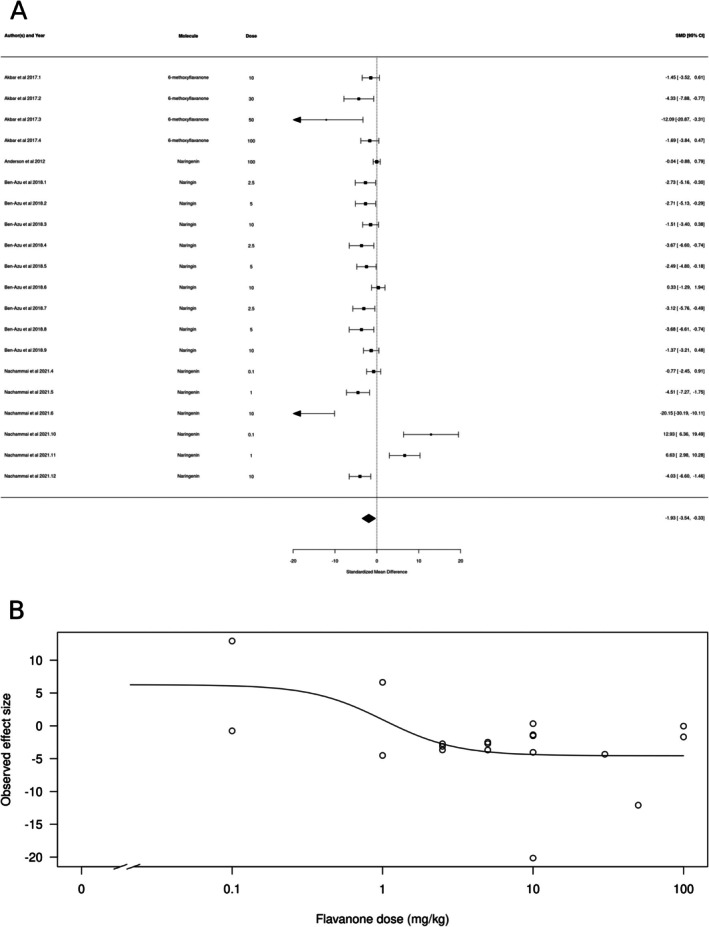
Subgroup analysis for flavanones. (A) Forest plot showing the results of 20 comparisons examining the effect of a flavanone on anxiety‐like behavior in animal tests. The figure shows the standardized mean difference (SMD) between control and flavanone‐treated groups with corresponding 95% confidence intervals in the individual comparison, based on a random‐effects model. A negative standardized mean difference (SMD) corresponds to decreased anxiety‐like behavior, while a positive SMD corresponds to increased anxiety‐like behavior after flavanone treatment. The overall effect size is denoted by the diamond symbol. (B) Estimated dose–response curve, based on the observed effect size (SMD) with increasing flavanone dose. Each point corresponds to a single comparison from the forest plot. The curve was obtained by fitting generalized log‐logistic 5‐parameter models, with dose as independent variable and observed effect size SMD as dependent variable.

Flavanones (*k* = 20) also appeared to produce an anxiolytic‐like effect (Figure 5A). The estimated average standardized mean difference based on the random‐effects model was SMD = −1.93 (95% CI: −3.54 to −0.33), interpreted as a small‐to‐large effect size. Therefore, the average outcome differed significantly from zero (*z* = −2.363, *p* = 0.018), suggesting an overall anxiolytic‐like effect. According to the Q‐test, the true outcomes appear to be highly heterogeneous (*Q*[df = 19] = 91.4232, *p* < 0.0001, *τ* ^2^ = 0.2743, *I* ^2^ = 89.61%). There is some evidence for a dose‐dependent effect, with higher doses producing slightly larger effects (Figure 5B), and a calculated ED50 of 1.02 ± 1.01 mg/kg (mean ± SEM).

**FIGURE 6:**
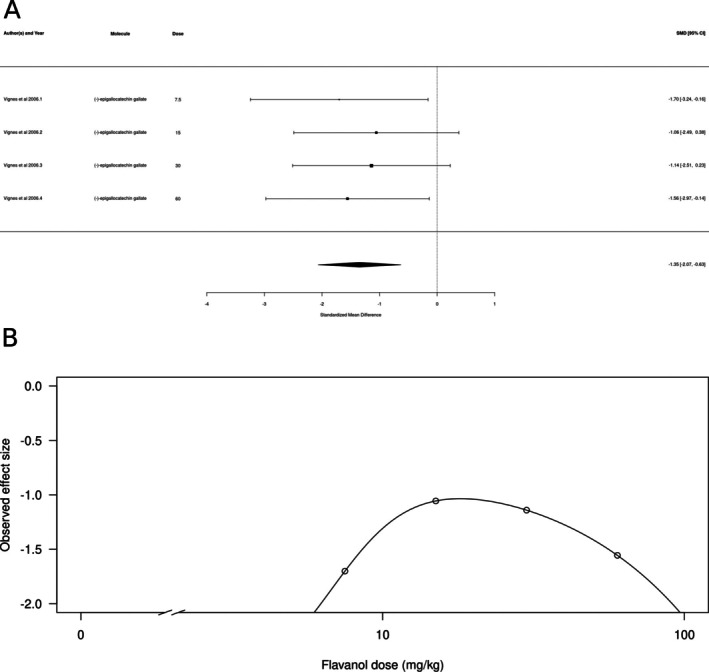
Subgroup analysis for flavanols. (A) Forest plot showing the results of four comparisons examining the effect of a flavanol on anxiety‐like behavior in animal tests. The figure shows the standardized mean difference (SMD) between control and flavanol‐treated groups with corresponding 95% confidence intervals in the individual comparison, based on a random‐effects model. A negative standardized mean difference (SMD) corresponds to decreased anxiety‐like behavior, while a positive SMD corresponds to increased anxiety‐like behavior after flavanol treatment. The overall effect size is denoted by the diamond symbol. (B) Estimated dose–response curve, based on the observed effect size (SMD) with increasing flavanol dose. Each point corresponds to a single comparison from the forest plot. The curve was obtained by fitting a 5‐parameter Brain‐Cousens model, with dose as an independent variable and observed effect size SMD as a dependent variable.

Flavanols (*k* = 4, represented only by (−)‐epigallocatechin gallate) also appeared to produce an anxiolytic‐like effect (Figure 6A). The estimated average standardized mean difference based on the random‐effects model was SMD = −1.35 (95% CI: −2.07 to −0.63), interpreted as a medium‐to‐large effect size. Therefore, the average outcome differed significantly from zero (*z* = −3.6756, *p* = 0.0002), suggesting an overall anxiolytic‐like effect. According to the Q‐test, the true outcomes appear to be highly homogeneous (*Q*[df = 3] = 0.3665, *p* = 0.912, *τ* ^2^ = 0, *I* ^2^ = 0.0%), as expected by an analysis that includes a single study. There is some evidence for a dose‐dependent hormetic effect, with lower and higher doses producing slightly larger effects (Figure 6B), and a calculated ED50 of 75.56 ± 0.11 mg/kg (mean ± SEM).

**FIGURE 7:**
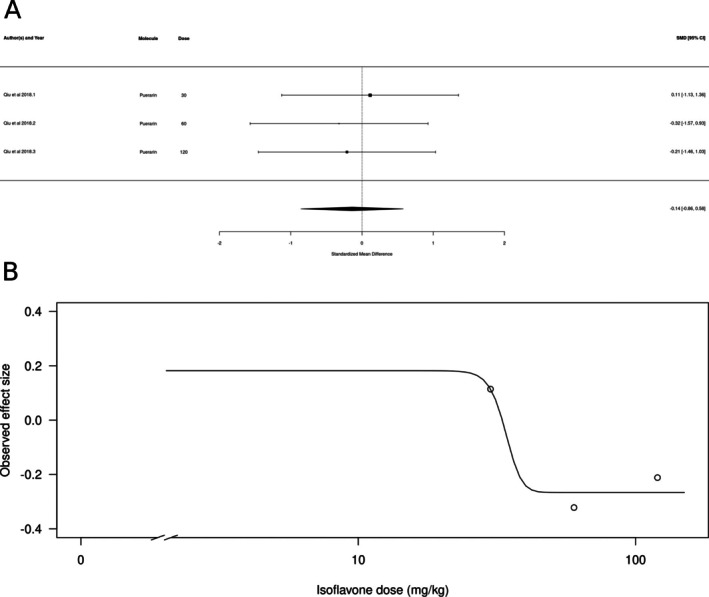
Subgroup analysis for isoflavones. (A) Forest plot showing the results of three comparisons examining the effect of an isoflavone on anxiety‐like behavior in animal tests. The figure shows the standardized mean difference (SMD) between control and isoflavone‐treated groups with corresponding 95% confidence intervals in the individual comparison, based on a random‐effects model. A negative standardized mean difference (SMD) corresponds to decreased anxiety‐like behavior, while a positive SMD corresponds to increased anxiety‐like behavior after isoflavone treatment. The overall effect size is denoted by the diamond symbol. (B) Estimated dose–response curve, based on the observed effect size (SMD) with increasing isoflavone dose. Each point corresponds to a single comparison from the forest plot. The curve was obtained by fitting generalized log‐logistic 5‐parameter models, with dose as an independent variable and the observed effect size SMD as the dependent variable.

Isoflavones (*k* = 3, represented only by puerarin) did not appear to produce anxiolytic‐ or anxiogenic‐like effects (Figure 7A). The estimated average standardized mean difference based on the random‐effects model was SMD = −0.3065 (95% CI: −0.98 to 0.36). Therefore, the average outcome did not significantly differ from zero (*z* = −0.8978, *p* = 0.3693). In concordance with the wide range of SMDs, the true outcomes appear to be highly heterogeneous (Q[df = 20] = 78.746, *p* < 0.0001, *τ* ^2^ = 1.889, *I* ^2^ = 83.17%). There is weak evidence for a dose‐dependent effect, with higher doses producing slightly larger effects (Figure 7B), but it was not possible to calculate an ED50.

**FIGURE 8:**
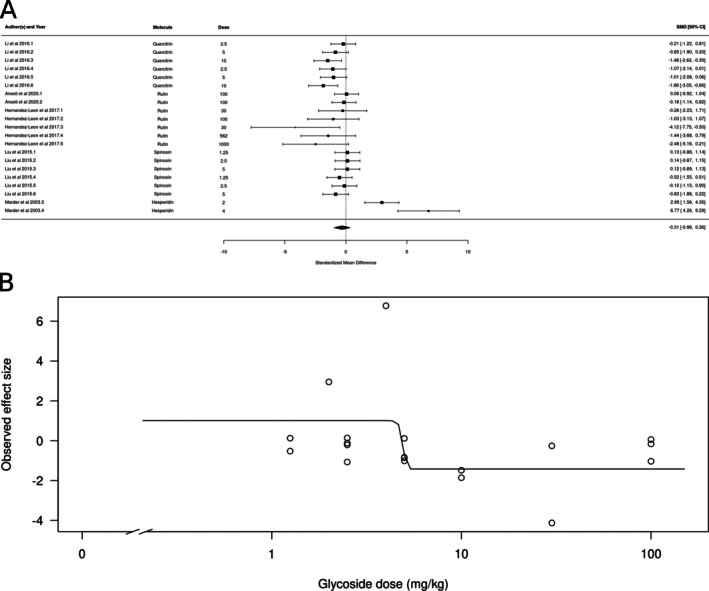
Subgroup analysis for glycoside derivatives. (A) Forest plot showing the results of 24 comparisons examining the effect of a glycoside derivative on anxiety‐like behavior in animal tests. The figure shows the standardized mean difference (SMD) between control and glycoside derivative‐treated groups with corresponding 95% confidence intervals in the individual comparison, based on a random‐effects model. A negative standardized mean difference (SMD) corresponds to decreased anxiety‐like behavior, while a positive SMD corresponds to increased anxiety‐like behavior after glycoside derivative treatment. The overall effect size is denoted by the diamond symbol. (B) Estimated dose–response curve, based on the observed effect size (SMD) with increasing glycoside derivative dose. Each point corresponds to a single comparison from the forest plot. The curve was obtained by fitting generalized log‐logistic 5‐parameter models, with dose as the independent variable and observed effect size SMD as the dependent variable.

Glycoside derivatives (*k* = 24) also did not appear to produce anxiolytic‐ or anxiogenic‐like effects (Figure 8A). The estimated average standardized mean difference based on the random‐effects model was SMD = −0.43 (95% CI: −1.06 to 0.16). Therefore, the average outcome did not significantly differ from zero (*z* = −1.45, *p* = 0.1466). According to the *Q*‐test, the true outcomes appear to be heterogeneous (*Q*[df = 23] = 84.09, *p* < 0.001, *τ* ^2^ = 1.7288, I^2^ = 80.65%). There is weak evidence for a dose‐dependent effect, with higher doses producing larger effects (Figure 8B), with an ED50 of 4.94 ± 0.34 mg/kg (mean ± SEM).

**FIGURE 9:**
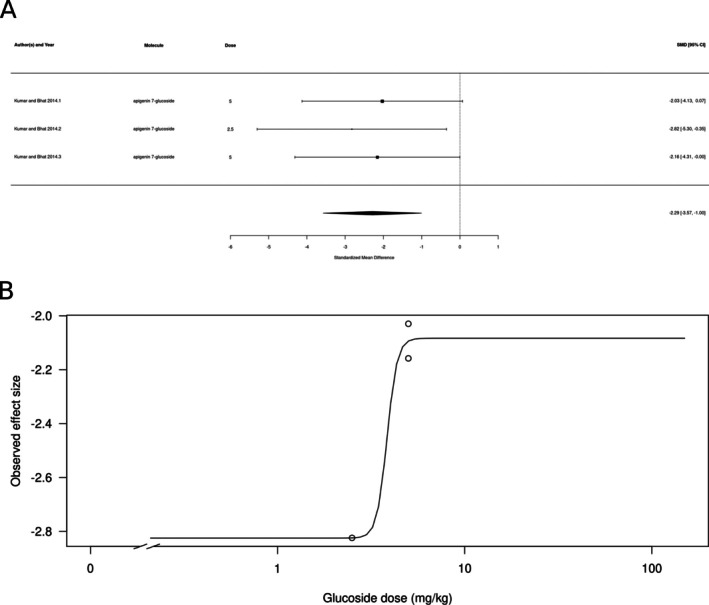
Subgroup analysis for glucoside derivatives. (A) Forest plot showing the results of 3 comparisons examining the effect of a glucoside derivative on anxiety‐like behavior in animal tests. The figure shows the standardized mean difference (SMD) between control and glucoside derivative‐treated groups with corresponding 95% confidence intervals in the individual comparison, based on a random‐effects model. A negative standardized mean difference (SMD) corresponds to decreased anxiety‐like behavior, while a positive SMD corresponds to increased anxiety‐like behavior after glucoside derivative treatment. The overall effect size is denoted by the diamond symbol. (B) Estimated dose–response curve, based on the observed effect size (SMD) with increasing glucoside derivative dose. Each point corresponds to a single comparison from the forest plot. The curve was obtained by fitting generalized log‐logistic 5‐parameter models, with dose as the independent variable and observed effect size SMD as the dependent variable.

Glucoside derivatives (*k* = 3, represented by apigenin 7‐glucoside, hesperidin, and tricin‐7‐O‐glucoside) appeared to not produce an anxiolytic‐like effect (Figure 9A). The estimated average standardized mean difference based on the random‐effects model was SMD = −1.16 (95% CI: −3.57 to 1.22), interpreted as a moderate effect size. Therefore, the average outcome did not significantly differ from zero (*z* = −0.9543, *p* = 0.34), suggesting no overall anxiolytic‐like effect. According to the *Q*‐test, the true outcomes appear to be heterogeneous (*Q*[df = 2] = 10.69, *p* = 0.048, *τ* ^2^ = 3.47, *I* ^2^ = 78.63%). There is some evidence for a dose‐dependent effect, with lower doses producing larger effects (Figure 9B), albeit with high variability: a calculated ED50 of 7.35 ± 36.49 mg/kg (mean ± SEM).

**FIGURE 10:**
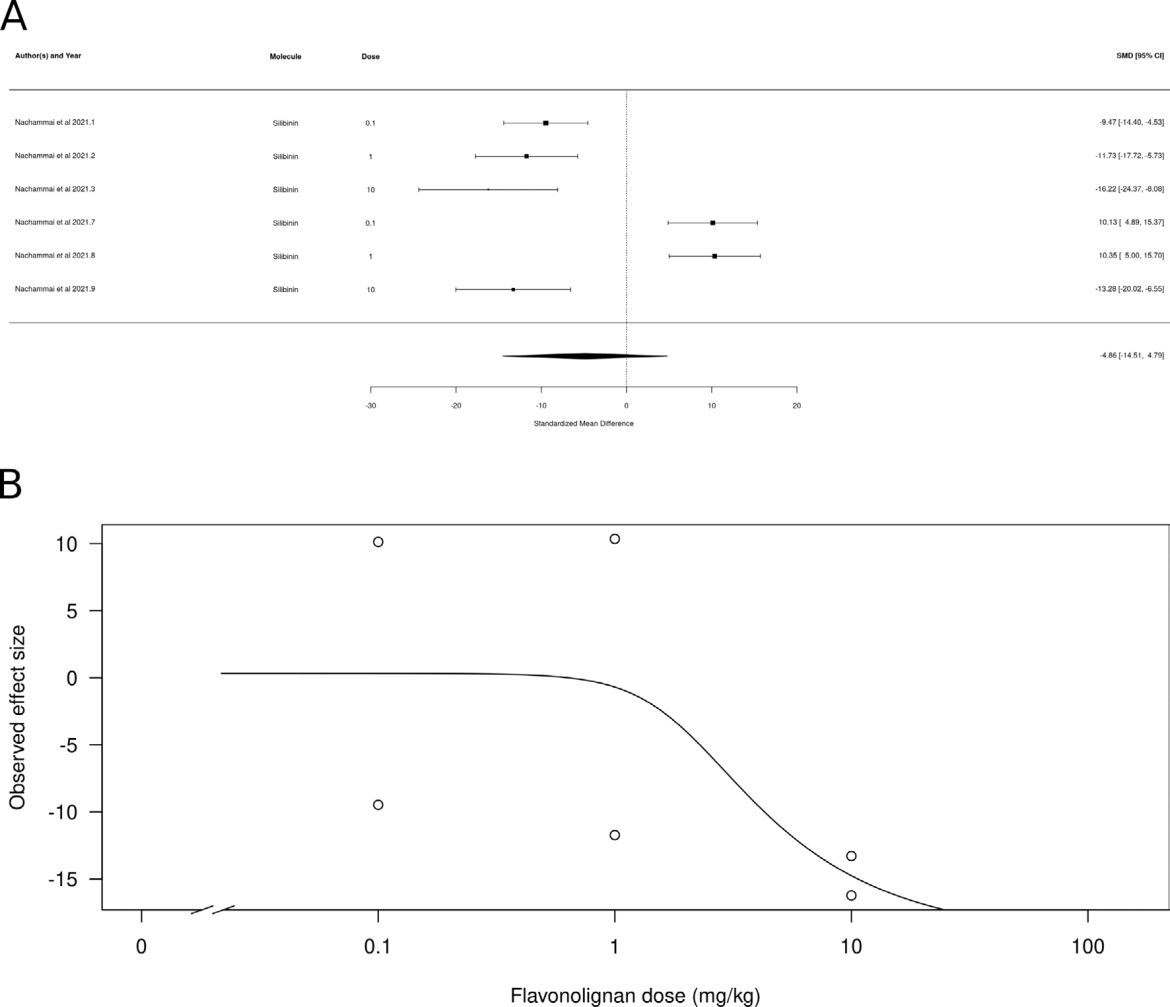
Subgroup analysis for flavonolignans. (A) Forest plot showing the results of 6 comparisons examining the effect of a flavonolignan on anxiety‐like behavior in animal tests. The figure shows the standardized mean difference (SMD) between control and flavonolignan‐treated groups with corresponding 95% confidence intervals in the individual comparisons, based on a random‐effects model. A negative standardized mean difference (SMD) corresponds to decreased anxiety‐like behavior, while a positive SMD corresponds to increased anxiety‐like behavior after flavonolignan treatment. The overall effect size is denoted by the diamond symbol. (B) Estimated dose–response curve, based on the observed effect size (SMD) with increasing flavonolignan dose. Each point corresponds to a single comparison from the forest plot. The curve was obtained by fitting generalized log‐logistic 5‐parameter models, with dose as an independent variable and observed effect size SMD as a dependent variable.

Finally, flavonolignans (*k* = 6, represented only by silibinin) did not appear to produce either anxiolytic‐ or anxiogenic‐like effects (Figure 10A). The estimated average standardized mean difference based on the random‐effects model was SMD = −4.86 (95% CI: −14.51 to 4.79). Therefore, the average outcome did not significantly differ from zero (*z* = −0.9867, *p* = 0.3238). According to the *Q*‐test, the true outcomes appear to be highly heterogeneous (Q[df = 5] = 81.86, *p* < 0.0001, *τ* ^2^ = 135.7513, *I* ^2^ = 96.8%). There is some evidence for a dose‐dependent effect, with higher doses producing larger effects (Figure 10B), albeit with high variability: a calculated ED50 of 4.04 ± 687.98 mg/kg (mean ± SEM).

## 4 Discussion

The present work presents a bibliometric analysis of the literature on the acute and chronic effects of natural and synthetic flavonoids on anxiety‐like behavior in animal tests, followed by a meta‐analysis of filtered data. Bibliometric analysis suggested a prolific field that can improve in terms of international collaboration; thematic analysis suggests that the field was highly focused on testing drug effects, with part of it focusing on mechanisms—especially neurobiological effects on oxidative stress and neuropathology—with few emerging themes. The meta‐analysis revealed a general anxiolytic‐like effect was found for these molecules, with a high heterogeneity in findings. Subgroup analyses found that acute treatment produced significant effects, while no evidence for chronic effects was found. Moreover, it was found that, for all molecule classes included in the study, only isoflavones, glycoside derivatives, and flavanolignans did not show evidence of an anxiolytic‐like effect. Finally, we found evidence for publication bias, suggesting a “file‐drawer effect.”

### 4.1 Mapping the Field With Bibliometric Analyses

Bibliometric analysis suggests that the field is highly concentrated on few research groups that are mostly located in the Global South, including Asian, African, Middle Eastern, and Latin American countries. These results are not surprising, considering that China and India are the top two research‐producing countries in the field of flavonoids, both in plant physiology (Li et al. 2023) and health (Perez‐Vizcaino and Fraga 2018). Li et al. (2023) attribute part of this trend to the rapid growth of agricultural research and production in these countries. International collaborations are still very incipient, with most research groups being formed by individuals from the same institution and a low proportion of multiple country publications. Improving international collaborations seems to be an important step forward in the field; however, the current framework for international collaboration policies still implies the construction of inequalities in capabilities and abilities, with a lack of reciprocity and agency (Martinez and Sá 2019; Pineda et al. 2019; Skupien and Rüffin 2019). Considering that most of the research on anxiolytic‐like effects of flavonoids tends to happen in the “Global South,” strategies to mitigate these inequalities are needed.

The analysis of trending topics suggests that the field is still very much dominated by exploratory research on the putative behavioral effects of flavonoids and their phytochemical characterization. This is indicated by the fact that the “motor themes” (those with high centrality and density in each field, being both well‐developed and important for the structure of the area) involve mainly areas of phytochemistry, including compound extraction and quantification methods. Moreover, the “core sources” for research on anxiolytic‐like effects of flavonoids include mainly journals from the area of pharmacology. Conversely, the basic themes (which involve themes that are important for a given research field, but are underdeveloped) include basic techniques in behavioral pharmacology (dose–response relationships, different behavioral methods), as well as general mechanisms of drug effect. This is in contrast with the multitude of known mechanisms of action of flavonoids, which include modulation of GABAergic receptors (Hanrahan et al. 2011; Wasowski and Marder 2012), monoamine metabolism (Carradori et al. 2016), and antioxidant properties (Hritcu et al. 2017). Niche and emerging or declining themes, which show either specialized or peripheral themes with low external ties to other themes, include the study of inflammation and neuropathology mechanisms, as well as the use of computational techniques. This suggests that the field, as a whole, could benefit from more mechanistic and confirmatory research.

### 4.2 Systematic Review and Meta‐Analysis

In contrast with the bibliometric analysis (which included all documents from the area retrieved from PubMed, not only those that met the inclusion criteria for the meta‐analysis), a systematic review of the experimental literature suggested a field that is more concerned with mechanisms. Of the 38 documents retained for the analysis, the majority also studied mechanisms of action, either indirectly (e.g., with competitive radioligand binding assays, or in silico molecular docking studies) or directly, by pre‐treating animals with different receptor antagonists. Most of these studies focused on GABAA receptors, analyzing either the orthosteric site for GABA or different allosteric sites. Indeed, we found a positive but small correlation between effect sizes and affinity for the GABAA receptor, although information for that last variable is lacking for most molecules in the dataset. A few studies (e.g., Ferreira et al. 2020; Liu et al. 2015) also investigated the participation of the serotonergic system.

The general meta‐analysis found evidence for an anxiolytic‐like effect in preclinical animal tests, albeit with high heterogeneity. The overall effect size is larger than that reported for clinical studies with flavonoids (Jia et al. 2022) or BZDs (Gomez et al. 2018) for the treatment of anxiety; this is probably due to the much higher heterogeneity in clinical studies that, by nature, lack experimental control. The high heterogeneity found in the general meta‐analysis prompted us to investigate its sources, focusing on treatment duration and molecule class.

Differences in the effects of treatment duration are highly relevant from a translational point of view, with implications for treatment regimens in humans. While BZDs are commonly prescribed for acute and subacute use, they produce pharmacological tolerance (Shinfuku et al. 2019) and important adverse effects (Liu et al. 2020), and the risk of addiction (Longo and Johnson 2000) increases with chronic treatment. Nonetheless, a meta‐analysis of clinical studies with flavonoids suggested that chronic treatment appears to produce better results (Jia et al. 2022). However, the fact that we found anxiolytic‐like effects of acute, but not chronic, treatment needs to be considered with caution, since it can be interpreted in terms of the number of studies, as only 4 of the 38 studies included in the meta‐analysis attempted chronic treatment. Further preclinical studies using flavonoids should incorporate chronic treatment, testing not only for the development of tolerance but also for the development of adverse effects, withdrawal‐like symptoms, and abuse potential.

We also found molecule class‐dependent anxiolytic‐like effects in the meta‐analysis. Subgroup analysis found significant effects of flavones, chalcones, flavonols, flavanones, flavanols, and glycoside derivatives. Most studies involved flavones and chalcones, which also had the larger effect sizes. 2′‐methoxy‐6‐methylflavone and 3‐hydroxy‐2′‐methoxy‐6‐methylflavone modulate the activity of GABAA receptors in subunit‐selective ways: the first appears to directly activate α2/γ2‐containing GABAA receptors, while the second acts as a positive allosteric modulation of the α2β2/3γ2L and direct activation of α4β2/3δ GABAA receptors (Karim et al. 2011, 2012). 5‐Methoxyflavone also appears to bind α2‐containing receptors (Shanmugasundaram et al. 2020). Chrysin, wogonin, 6‐methylapigenin, luteonin, and 5,7,2′‐trihydroxy‐6,8‐dimethoxyflavone all appear to have affinity for the central BZD binding site (Wasowski and Marder 2012). Apigenin, on the other hand, appears to act on GABAA receptors either as an inverse agonist or as an antagonist at the central BZD binding site (Avallone et al. 2000; Dekermendjian et al. 1999; Zanoli et al. 2000), and inspection of the forest plot in Figure 2A suggests that this molecule does not have a clear anxiolytic‐like effect in animal tests. There is also some evidence for a participation of the central BZD site in the effects of chalcones, at least in zebrafish (Ferreira et al. 2020, 2021; Santos Oliveira et al. 2024). While we found no association between affinity for the GABAA receptor and the effect size of the meta‐analysis, these results are limited by the very small number of molecules with empirically derived affinities, as well as by the fact that GABAA receptors show different allosteric sites that might be modulated by flavonoids (Wasowski and Marder 2012). On the other hand, flavones and chalcones also appear to act at other targets, including monoaminergic neurotransmission (Carradori et al. 2016) and redox balance (Hritcu et al. 2017). With few exceptions, most of the work on the effects of flavones and chalcones on anxiety‐like behavior did not explicitly test the hypothesis of a mediation by either of these systems.

Interestingly, in the case of chalcones, the dose–response curve from the meta‐analysis suggested a hormetic effect, with lower and higher doses producing a more prominent anxiolytic‐like effect. The flavanones naringenin, naringin, and 6‐methoxyflavanone; the flavanol (−)‐epigallocatechin gallate; and the glucoside derivative apigenin 7‐glucoside all appeared to decrease anxiety‐like behavior. (−)‐epigallocatechin gallate appears to produce a hormetic dose–response profile, and the effects of apigenin 7‐glucoside appear to decrease with increasing dose. Hormesis is defined as a biphasic response, with stimulatory and inhibitory responses that can be either be mediated by a single receptor recruiting different downstream signaling mechanisms, by different receptors to which the molecule has different affinities, or by different targets at different levels of a signaling pathway (Calabrese 2013; Calabrese and Baldwin 2002). There is some evidence for both GABAergic and serotonergic mechanisms in mediating the anxiolytic‐like effects of chalcones (Ferreira et al. 2019, 2020; Mendes et al. 2023); however, since assessing hormetic mechanisms implicates blocking receptors at both the inhibitory and stimulatory doses of the chalcone (Calabrese 2013), currently there is no evidence supporting any specific hormetic mechanism for these molecules.

The flavonols kaempferol, quercetin, and myricetin also showed evidence for an anxiolytic‐like effect. The mechanisms of action of these flavanols are uncertain; intracerebroventricular injection of kaempferol reduces anxiety‐like behavior in male rats by a GABAA receptor‐dependent mechanism (Zarei et al. 2021). However, intraperitoneal injections of these three flavanols failed to affect anxiety‐like behavior, while administration *per os* decreased it, suggesting that these flavonols are pro‐drugs that are metabolized before they reach the brain (Vissiennon et al. 2012).

Interestingly, no effects were found for isoflavones, a class of molecules that is also classified as a phytoestrogen. The isoflavone content of the diet has been associated with increased anxiety‐like behavior in male rats (Hartley et al. 2003), but in non‐gonadectomized female rats, isoflavone supplementation has an anxiolytic‐like effect (Friedman and Frye 2011). In a meta‐analysis of the outcomes of dietary supplementation of flavonoids in women, isoflavones failed to produce an effect on anxiety outcomes (Jia et al. 2022). Again, care must be taken in the extrapolation of the results from the present meta‐analysis, given that only one study using a single isoflavone, puerarin, was included in the analysis, and the study used only acute treatment.

### 4.3 Study Quality and Risk of Bias

An important limitation of our meta‐analysis is that most of the studies showed an overall risk of bias representative of some concern. In fact, only two studies (Costa et al. 2025; German‐Ponciano et al. 2020) show low risk of bias. None of the studies were pre‐registered, and therefore protocols are unavailable; moreover, it is usually unclear, from the description of methods, whether or not caregivers, experimenters, and/or outcome assessors were blinded, and if and how randomization was present. These limitations greatly decrease the reproducibility of the preclinical experiments included in the meta‐analysis (Spanagel 2022). The small sample sizes reported in the studies, which is typical of preclinical research, also suggest that studies were underpowered, which could contribute to the negative findings of some classes; while this has not been assessed in the risk of bias tool employed in this paper (Hooijmans et al. 2014), most of the studies did not report sample size calculation, and therefore it is difficult to judge whether or not this is the case. Finally, more than half of the studies did not use standardized compounds, an important condition of reproducibility in natural products research (Izzo et al. 2016). Since the majority of studies which did not use standardized compounds reported data on isolation, purification, and analysis of the compounds, this is mostly justified by the focus of phytochemical research in finding *novel* compounds which can have anxiolytic activity. Nonetheless, some studies did not report analytic steps, or used fractions which are likely to contain other molecules than that of interest, which should be avoided whenever possible.

### 4.4 Recommendations for Future Research

Overall, the effects of flavonoids in preclinical behavioral research appear to indicate a class‐dependent effect, more evident at acute than chronic treatments, that are hypothesized to be mediated by GABAergic and/or 5‐HTergic mechanisms, although hypothesis testing of mechanisms is still weak. As a result, there are still many open questions in preclinical research of the putative anxiolytic‐like effects of flavonoids to be answered before these molecules are advanced in the drug development pipeline. Most of the outstanding questions are related to basic pharmacological and psychopharmacological research. Based on the bibliometric analysis, as well as, on the systematic review and meta‐analysis, we recommend that:

1. *The field needs to further explore mechanistic hypothesis‐driven research*: While in the initial stages of drug development exploratory research (including the establishment of dose–response curves, ligand binding and/or molecular docking assays, and the use of behavioral test batteries) is crucial, once these are established the search for mechanisms is fundamental to establish a good evidence base (Crabbe and Morris 2004; Stegenga 2022). This includes not only investigating classic pathways, such as activation of the GABA_A_ receptor, but also the roles of oxidative stress, neurosteroidogenesis, and serotonergic signaling, all of which have been shown to be targeted by flavonoids.
2. *In order to do so, increasing collaboration between groups could be important*: Mechanistic research usually involves the use of multiple techniques, from ligand binding, molecular docking, and electrophysiology and/or signal transduction biochemistry, to molecular biology and behavioral pharmacology assays. Not all research groups will be able to tackle all the questions that are important for mechanistic research—especially considering that most of the groups currently working in the field come from the Global South. Thus, increasing collaboration between research groups could be important to move the field toward more mechanistic, hypothesis‐driven research.
3. *Improving reproducibility and increasing power is paramount*: Most of the documents found in the meta‐analysis rely on very small sample sizes, typical of exploratory research, which can lead to inflated effect sizes and might be responsible for the high heterogeneity. As research in the field advances, sample sizes will need to be increased in order to approach true effects and reduce heterogeneity. Moreover, steps towards increasing reproducibility are also direly needed (Begley and Ioannidis 2015; Richter et al. 2009); the analysis of risk of bias showed that most studies did not report whether (or how) blinding and randomization were achieved. Again, multi‐site collaborations could help, as introducing heterogeneity in these studies (with each laboratory covering a portion of the final sample size) appears to increase reproducibility while decreasing the need for a single group bearing the weight of large sample sizes (Drude et al. 2022; Voelkl et al. 2018).

Flavonoids can represent a very promising lead in anxiolytic research, a field that is in dire need of novel molecules that can be translated from preclinical to clinical research. However, for that potential to be realized, research practices in the field need to advance. We hope that, in the near future, the field of flavonoid research reaches the maturity it needs.

## Author Contributions

**Jhennify Albuquerque Machado:** conceptualization, data curation, formal analysis, investigation, methodology, writing – original draft. **Danilo Brandão Araújo:** conceptualization, data curation, formal analysis, investigation, methodology, writing – original draft. **Monica Lima‐Maximino:** conceptualization, methodology, writing – original draft. **Diógenes Henrique de Siqueira‐Silva:** conceptualization, methodology, writing – original draft. **Bernardo Tomchinsky:** conceptualization, methodology, writing – original draft. **Jonathan Cueto‐Escobedo:** conceptualization, methodology, writing – original draft. **Juan Francisco Rodríguez‐Landa:** conceptualization, methodology, writing – original draft. **Caio Maximino:** conceptualization, data curation, formal analysis, funding acquisition, methodology, project administration, supervision, validation, writing – original draft.

## Conflicts of Interest

The authors declare no conflicts of interest.

## Supporting information


**Data S1:** PRISMA Checklist.

**Figure S1:** Bibliometric analysis of the field of preclinical research on the anxiolytic‐like effects of flavonoids in animal tests. (A) The changes in annual publications from 1994 to 2024. (B) Country scientific production and collaboration status; number of publications per country, either as single‐country publications (blue, SCP) or multiple country publications (red, MCP). (C) Keyword co‐occurrence network and clusters identified with bibliometrix. Each node represents a keyword that meet the filtering thresholds; node size is correlated with their publication numbers and the curved line represents the co‐occurrence relationship between keywords. (D) Thematic map, by density and centrality, based on author keywords.

**Figure S2:** Risk of bias assessment rating results. (A) Assessment of individual studies; colors code for risk ratings for each guiding question in SYRCLE's risk of bias tool (Hooijmans et al. 2014). (B) Summary of ratings for all studies; the four ratings are illustrated by percentage.

**Figure S3:** Forest plot showing the results of the overall meta‐analysis, with 183 comparisons examining the effect of a flavonoid on anxiety‐like behavior in animal tests. The figure shows the standardized mean difference (SMD) between control and flavonoid‐treated groups with corresponding 95% confidence intervals in the individual comparison, based on a random‐effects model. A negative standardized mean difference (SMD) corresponds to decreased anxiety‐like behavior, while a positive SMD corresponds to increased anxiety‐like behavior after flavonoid treatment. The overall effect size is denoted by the diamond symbol.

**Figure S4:** Funnel plot for the general meta‐analysis. Each plotted point represents the standardized mean difference (SMD) and the square root of the sample size between controls and flavonoid‐treated animals for a single comparison. The vertical line represents the average SMD of −1.457 found in the meta‐analysis. The asymmetry of the data points toward the left of this line is suggestive of publication bias.

**Figure S5:** Forest plot showing the results of the subgroup meta‐analysis for acute treatment, with 161 comparisons examining the effect of a flavonoid on anxiety‐like behavior in animal tests in acute treatment. The figure shows the standardized mean difference (SMD) between control and flavonoid‐treated groups with corresponding 95% confidence intervals in the individual comparison, based on a random‐effects model. A negative standardized mean difference (SMD) corresponds to decreased anxiety‐like behavior, while a positive SMD corresponds to increased anxiety‐like behavior after flavonoid treatment. The overall effect size is denoted by the diamond symbol.

**Figure S6:** Forest plot showing the results of the subgroup meta‐analysis for chronic treatment, with 22 comparisons examining the effect of a flavonoid on anxiety‐like behavior in animal tests in chronic treatment. The figure shows the standardized mean difference (SMD) between control and flavonoid‐treated groups with corresponding 95% confidence intervals in the individual comparison, based on a random‐effects model. A negative standardized mean difference (SMD) corresponds to decreased anxiety‐like behavior, while a positive SMD corresponds to increased anxiety‐like behavior after flavonoid treatment. The overall effect size is denoted by the diamond symbol.

**Table S1:** Characteristics of the studies included in the metanalysis.

## Data Availability

Data for this work can be found at https://github.com/lanec‐unifesspa/plantasmedicinais/tree/main/flavonoides.
