# Supplementary figures and images for: Flavonoids as Anxiolytics in Animal Tests: Systematic Review, Meta‐Analysis, and Bibliometrical Analysis
Source: Phytother Res. 2025 Oct 29;40(1):77–99. doi: 10.1002/ptr.70060 (PMC12796049; doi:10.1002/ptr.70060)

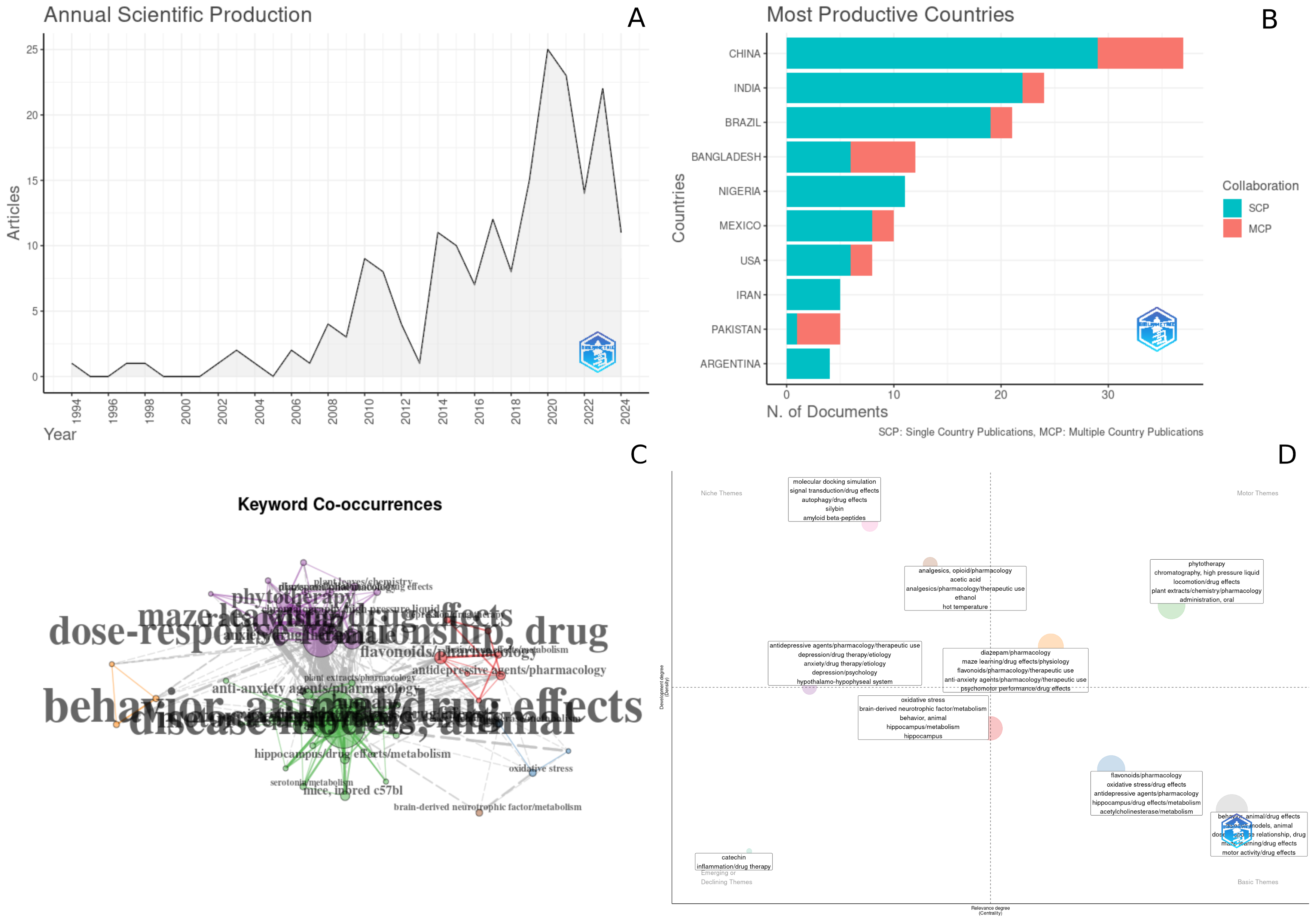

Supplement: Supplementary file 2 — Figure S1: Bibliometric analysis of the field of preclinical research on the anxiolytic‐like effects of flavonoids in animal tests. (A) The changes in annual publications from 1994 to 2024. (B) Country scientific production and collaboration status; number of publications per country, either as single‐country publications (blue, SCP) or multiple country publications (red, MCP). (C) Keyword co‐occurrence network and clusters identified with bibliometrix. Each node represents a keyword that meet the filtering thresholds; node size is correlated with their publication numbers and the curved line represents the co‐occurrence relationship between keywords. (D) Thematic map, by density and centrality, based on author keywords. [file PTR-40-77-s001.tiff]

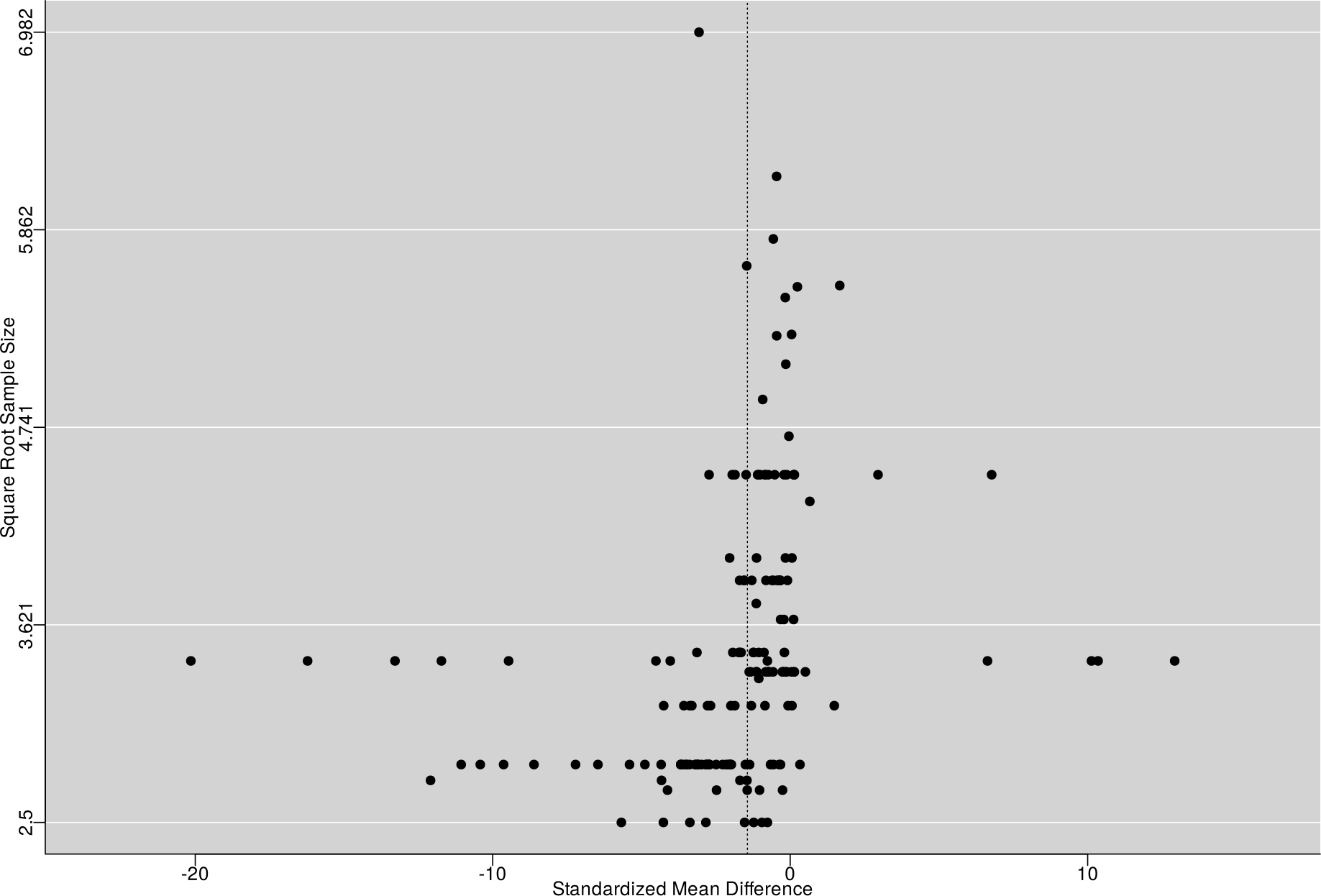

Supplement: Supplementary file 4 — Figure S3: Forest plot showing the results of the overall meta‐analysis, with 183 comparisons examining the effect of a flavonoid on anxiety‐like behavior in animal tests. The figure shows the standardized mean difference (SMD) between control and flavonoid‐treated groups with corresponding 95% confidence intervals in the individual comparison, based on a random‐effects model. A negative standardized mean difference (SMD) corresponds to decreased anxiety‐like behavior, while a positive SMD corresponds to increased anxiety‐like behavior after flavonoid treatment. The overall effect size is denoted by the diamond symbol. [file PTR-40-77-s003.tiff]
